# Medical conditions of primary care patients with documented cannabis use and cannabis use disorder in electronic health records: a case control study from an academic health system in a medical marijuana state

**DOI:** 10.1186/s13011-022-00467-1

**Published:** 2022-05-08

**Authors:** Howard Padwa, David Huang, Larissa Mooney, Christine E. Grella, Darren Urada, Douglas S. Bell, Brittany Bass, Anne E. Boustead

**Affiliations:** 1 Department of Psychiatry and Biobehavioral Sciences, University of California, 11075 Santa Monica Boulevard, Suite 200, Los Angeles, CA 90025 USA; 2 Division of General Internal Medicine, Department of Medicine, University of California, Los Angeles, USA; 3 Clinical and Translational Science Institute, University of California, Los Angeles, USA; 4 School of Government & Public Policy, University of Arizona, Tucson, USA

**Keywords:** Cannabis, Comorbidities, Primary Care, Co-occurring disorders, Cannabis legalization

## Abstract

**Background:**

Approximately 3.8% of adults worldwide have used cannabis in the past year. Understanding how cannabis use is associated with other health conditions is crucial for healthcare providers seeking to understand the needs of their patients, and for health policymakers. This paper analyzes the relationship between documented cannabis use disorders (CUD), cannabis use (CU) and other health diagnoses among primary care patients during a time when medical use of marijuana was permitted by state law in California, United States of America.

**Methods:**

The study utilized primary care electronic health record (EHR) data from an academic health system, using a case–control design to compare diagnoses among individuals with CUD/CU to those of matched controls, and those of individuals with CUD diagnoses with individuals who had CU otherwise documented. Associations of documented CU and CUD with general medical conditions and health conditions associated with cannabis use (both medical and behavioral) were analyzed using conditional logistic regression.

**Results:**

Of 1,047,463 patients with ambulatory encounters from 2013–2017, 729 (0.06%) had CUD diagnoses and 3,731 (0.36%) had CU documented in their EHR. Patients with documented CUD and CU patients had significantly (*p* < 0.01) higher odds of most medical and behavioral diagnoses analyzed. Compared to matched controls, CUD-documented patients had highest odds of other substance use disorders (OR = 21.44: 95% CI 9.43–48.73), any mental health disorder (OR = 6.99; 95% CI 5.03–9.70) social anxiety disorder (OR = 13.03; 95% CI 2.18–77.94), HIV/AIDS (OR = 7.88: 95% CI 2.58–24.08), post-traumatic stress disorder (OR = 7.74: 95% CI 2.66–22.51); depression (OR = 7.01: 95% CI 4,79–10.27), and bipolar disorder (OR = 6.49: 95% CI 2.90–14.52). Compared to matched controls, CU-documented patients had highest odds of other substance use disorders (OR = 3.64; 95% CI 2.53–5.25) and post-traumatic stress disorder (OR = 3.41; 95% CI 2.53–5.25). CUD-documented patients were significantly more likely than CU-documented patients to have HIV/AIDS (OR = 6.70; 95% CI 2.10–21.39), other substance use disorder (OR = 5.88; 95% CI 2.42–14.22), depression (OR = 2.85; 95% CI 1.90–4.26), and anxiety (OR = 2.19: 95% CI 1.57–3.05) diagnoses.

**Conclusion:**

The prevalence of CUD and CU notation in EHR data from an academic health system was low, highlighting the need for improved screening in primary care. CUD and CU documentation were associated with increased risk for many health conditions, with the most elevated risk for behavioral health disorders and HIV/AIDS (among CUD-documented, but not CU-documented patients). Given the strong associations of CUD and CU documentation with health problems, it is important for healthcare providers to be prepared to identify CU and CUD, discuss the pros and cons of cannabis use with patients thoughtfully and empathically, and address cannabis-related comorbidities among these patients.

## Background

Cannabis is the most widely used psychoactive substance after alcohol and tobacco, with approximately 3.8% of the world’s adult population having used the drug in the past year [1]. The perceived harms associated with cannabis are decreasing [2, 3] while belief in its potential health benefits is increasing [3–5] and countries across the world are beginning to legalize cannabis for medical and/or non-medical use [6]. These trends make it likely that cannabis use will become increasingly common in the future [2, 7, 8]. Cannabis can have serious impacts on health [9–14], and patients often use the drug instead of other prescription medications to manage their health [13]. The purpose of this paper is to better understand the relationship between cannabis use, as documented in electronic health records (EHR), and other health diagnoses among primary care patients in a time when state law allowed for medical cannabis use, but not non-medical cannabis use.

Understanding the associations between cannabis use, medical conditions, and behavioral health can equip practitioners to more effectively identify patients who use cannabis and address the possible impacts the drug may have on their health. Currently, little is known about how often cannabis use is identified among primary care patients in health systems, or the degree to which it is associated with medical and psychiatric diagnoses among primary care populations. EHR data can be used to address these questions. For example, Campbell and colleagues [15] analyzed EHR data from community clinics in Oregon, California, and Washington State between 2012 and 2016, and found that primary care patients were more likely to have a cannabis use disorder (CUD), but not cannabis use without a disorder (CU), documented in EHRs if they had psychiatric diagnoses. Lapham and colleagues [16] analyzed data from a large integrated healthcare system in Washington State from 2015 to 2016, and found that mental health disorders, depression symptoms, tobacco use, unhealthy alcohol use, illicit drug use, and substance use disorders were associated with increased cannabis use. Matson and colleagues [17] recently analyzed EHR data from a large integrated healthcare system in Washington State from 2017 to 2018 to measure the prevalence of documented medical cannabis use and its association with health conditions for which cannabis use could be potentially beneficial or harmful. They found that patients who had documented medical use of cannabis had higher prevalence of diagnoses for both conditions that could be adversely impacted or helped by cannabis use when compared to non-medical users and non-users [17].

Campbell and colleagues’ data was collected from a mix of states that allowed for both medical (California at the time of data collection) and non-medical cannabis use (Oregon, Washington), while Matson and colleagues’ data was collected only from Washington. To our knowledge, no published research has used EHR data to examine the prevalence of cannabis use or the association of cannabis with health diagnoses in places where cannabis is only legal for medical use. It is important to analyze data from samples in locations that have different cannabis policies because cannabis’ legal status can influence who decides to use the drug, how frequently they use it, and the potency of the cannabis they consume [18–22]. Policy contexts also affect cannabis pricing, access, marketing, and social acceptability [23], which in turn can lead to differences in cannabis use and its consequences. The legal status of cannabis may influence patients’ willingness to disclose their use to their physician when they seek treatment for a health problem leading to differences in how frequently it is documented in EHRs, and it can also impact the degree to which cannabis use may have negative social or legal consequences [5].

The goal of this paper is to complement the work of Campbell et al. [15] and Matson et al. [17] by analyzing EHR data from California between 2013 and 2017, when cannabis was only legal for medical use. As of May 2021, 18 states in the United States (U.S.) [24] and countries across the world—including the United Kingdom [25], Australia [26], and many nations in continental Europe [27] and South America [28] — allowed for medical cannabis use, but not adult (non-medical) cannabis use. Findings from this study can be used to inform clinical practice in these places, and other jurisdictions that may allow medical cannabis use—but not non-medical cannabis use—in the future.

The paper has two aims: (1) to measure the prevalence of documented cannabis use disorder (CUD) and cannabis use (CU) in a large health system’s EHR; and (2) to determine the odds that patients with documented CUD and CU had general co-occurring medical conditions and conditions known to be associated with cannabis use.

## Methods

### Study design

The study utilized EHR data from the Internal Medicine and Family Medicine departments of the University of California, Los Angeles Health System utilizing Epic/Clarity software. To determine the odds that individuals with CUD/CU documentation had specific health conditions, the study utilized a case–control design, comparing diagnoses among individuals with CUD/CU documentation to those of matched controls, and those of individuals with CUD diagnoses to those of individuals with documented CU.

### Study sample

The study sample was drawn from 1,047,463 unique primary care patients aged 18 or older who had ambulatory care encounters documented in the health system EHR between January 1, 2013 and September 1, 2017. Sample patients had relatively high socio-economic status, as the median household income of health system patients’ neighborhoods was over $84,000, and over 64% of the patients had private insurance. Patients with CUD were identified with International Classification of Diseases, Tenth Revision (ICD-10) code F12 (cannabis-related disorders). Patients in the CU documentation group were identified by text searches of EHR social history notes, as has been done in other studies [15, 29]. Patients were considered part of the CU group if there was mention of “cannabis,” misspelled variations of cannabis (“cannibis,” “canbis”, “canibis”) or common colloquial equivalents (“marijuana”, “pot”, “weed”, “grass”) in EHR notes but there was no CUD diagnosis. In the event patients met both CUD and CU criteria, they were considered CUD patients. No exclusion criteria were used in the selection of case patients. Patient controls were identified and matched to the case patients by sex (male/female), race/ethnicity (White, Black, Hispanic, Asian/Pacific Islander, Other/Unknown), age (18–29, 30–39, 40–49, 50–59, 60–69, 70 +), and first encounter year within the EHR system.

### Measures

Diagnoses were identified by their International Classification of Diseases, Tenth Revision (ICD-10) codes in EHRs (see Appendix for the ICD-10 codes utilized). General co-occurring medical conditions examined included cancer, nervous system disease, circulatory system disease, respiratory disease, digestive system diseases, liver disease, diseases of the musculoskeletal system, HIV/AIDS, sexually transmitted diseases other than HIV/AIDS, mental health disorders, alcohol use disorders, tobacco use disorders, and substance use disorders other than those related to alcohol, cannabis, and tobacco.

Conditions known to be associated with cannabis were based on the National Academies of Sciences, Engineering and Medicine review of the health effects of cannabis and cannabinoids [13]. These conditions included cancer, respiratory disease, chronic obstructive pulmonary disease (COPD), ischemic heart disease (an indicator for myocardial infarction and ischemic stroke), obstructive sleep apnea, multiple sclerosis, Tourette Syndrome, testicular cancer, chronic pain, fibromyalgia, HIV/AIDS, schizophrenia/psychotic disorders, depression, anxiety, bipolar disorder, social anxiety disorder, post-traumatic stress disorder (PTSD), alcohol use disorders, and other substance use disorders. See the Appendix for the ICD-10 codes used to identify these conditions.

### Analyses

The odds that patients with CU or CUD documentation had had various physical, mental health, and substance use disorder diagnoses were calculated using conditional logistic regression models that utilized pairs (each with one case and one control) as strata. First, a simple conditional logistic regression model was applied to each health condition, in which the presence of each diagnosis was included as the dependent outcome variable and the three groups (CU documentation, CUD documentation, controls) were included as the independent covariates. Using the control group as the reference group, the odds ratios (ORs) of the CU-documented and CUD-documented groups were estimated and statistically tested. A post-hoc analysis was also conducted to estimate and test the OR of CUD documentation compared to CU documentation for each condition controlling for alcohol and tobacco use disorders. ORs were then estimated and tested. A sequentially rejective test procedure [30] was then applied to control for type-one error for multiple comparisons that could emerge due to the large number of tests conducted, and alpha levels were adjusted accordingly at the *p* = 0.001 level. All analyses were conducted using SAS 9.4 analytic software. All study procedures and analyses were approved by the University of California, Los Angeles Institutional Review Board.

## Results

### Sample characteristics

Table 1 provides an overview of sample characteristics. Overall, the sample was majority male, White, and aged 18–39.

**Table 1:** Sample characteristics

	**Cannabis Use Disorder (CUD)-Documented (*N* = 729)**	**Cannabis Use, (no disorder) (CU)-Documented (*N* = 3,741)**	**Matched Controls** **(4,470)**	**Total** **(8,940)**
**Sex**				
Male	556 (77.6%)	2,361 (63.1%)	2,915 (65.2%)	5,832 (65.2%)
Female	173 (23.7%)	1,380 (36.9%)	1,555 (34.8%)	3,108 (34.8%)
**Race/Ethnicity**				
White	492 (67.5%)	2,406 (64.3%)	2,880 (64.4%)	5,778 (64.6%)
Black	48 (6.6%)	223 (6.0%)	270 (6.0%)	541 (6.1%)
Hispanic	41 (5.6%)	157 (4.2%)	195 (4.4%)	393 (4.4%)
Asian/Pacific Islander	25 (3.4%)	163 (4.4%)	202 (4.5%)	390 (4.4%)
Other/Unknown	123 (16.9%)	791 (21.1%)	922 (20.6%)	1,836 (20.5%)
**Age**				
18–29	256 (35.1%)	902 (24.1%)	1,158 (25.9%)	2,316 (25.9%)
30–39	157 (21.5%)	969 (25.9%)	1,132 (25.3%)	2,258 (25.3%)
40–49	97 (13.3%)	562 (15.0%)	651 (14.6%)	1,310 (14.7%)
50–59	88 (12.1%)	512 (13.7%)	604 (13.5%)	1,204 (13.5%)
60–69	84 (11.5%)	537 (14.4%)	606 (13.6%)	1,227 (13.7%)
70 +	47 (6.4%)	259 (6.9%)	319 (7.1%)	625 (7.0%)

### CU and CUD documentation

Of 1,047,463 patients, 4,470 had CUD diagnoses and/or a mention of cannabis in their social history notes; 729 (0.06%) had CUD diagnoses, and 3,741 (0.36%) were in the CU-documented group.

### Unadjusted odds of diagnoses

Table 2 includes the unadjusted prevalence of medical diagnoses among patients in the CUD-documented, CU-documented, and matched control groups.

**Table 2:** Unadjusted prevalence of diagnoses

	**Cannabis Use Disorder (CUD) – Documented *N* = 729**	**Cannabis Use** **(no disorder) (CU)-documented *N* = 3,741**	**Matched Controls** ***N* = 4,470**
**General Medical Conditions**			
Cancer	20.6%	22.1%	15.4%
Diabetes Mellitus	11.8%	7.9%	6.4%
Nervous System Disease	64.5%	52.2%	37.5%
Sleep Disorders	34.7%	23.2%	12.1%
Circulatory System Disease	45.7%	38.7%	29.7%
Respiratory Disease	63.0%	53.9%	43.3%
Digestive System Disease	58.2%	49.1%	34.2%
Liver Disease	12.6%	8.4%	5.2%
Musculoskeletal Disease	66.9%	62.3%	51.0%
HIV/AIDS	3.7%	0.8%	0.8%
Sexually Transmitted Disease	7.1%	3.9%	1.8%
Other Than HIV/AIDS			
Mental Health Disorders	71.1%	44.4%	21.9%
Alcohol Use Disorder	26.2%	6.5%	2.6%
Tobacco Use Disorder	36.6%	14.5%	4.9%
Substance Use Disorder (other than alcohol, cannabis, tobacco)	44.7%	13.8%	4.5%
**Conditions Known To Be Associated With Cannabis**			
Respiratory Disease	63.0%	53.9%	43.3%
COPD	26.2%	18.7%	13.1%
Cancer	20.6%	22.1%	15.4%
Ischemic Heart Disease	11.8%	7.3%	4.9%
Obstructive Sleep Apnea	7.5%	6.6%	3.5%
Multiple Sclerosis	0.8%	0.7%	0.2%
HIV/AIDS	3.7%	0.8%	0.8%
Tourette Syndrome	0.0%	0.1%	0.0%
Testicular Cancer	0.5%	0.2%	0.3%
Chronic Pain	70.0%	64.7%	52.5%
Schizophrenia/Psychotic Disorder	6.6%	1.7%	0.8%
Depression	50.3%	26.3%	12.4%
Anxiety	60.1%	36.3%	16.8%
Bipolar Disorder	11.1%	4.2%	1.5%
Social Anxiety Disorder	2.9%	0.6%	0.4%
Post-Traumatic Stress Disorder	7.4%	1.8%	0.6%
Alcohol Use Disorder	26.2%	6.5%	2.6%
Substance Use Disorder (other than cannabis, alcohol, tobacco)	44.7%	13.8%	4.5%

The CUD-documented group had higher unadjusted prevalence of most general medical conditions than the CU-documented and matched controls groups, and CU-documented patients had higher unadjusted prevalence of most conditions when compared to matched controls. The most pronounced differences in unadjusted prevalence were for any mental health disorders (71.1% of the CUD-documented group, 44.4% of the CU-documented group, 21.9% of matched controls), other substance use disorders (44.7% CUD-documented, 13.8% CU-documented, 4.5% matched controls), tobacco use disorder (36.6% CUD-documented, 14.5% CU-documented, 4.9% matched controls), and alcohol use disorder (26.2% CUD, 6.5% CU, 2.6% matched controls). Among diagnoses for conditions known to be associated with cannabis, there was a similar trend, with prevalence being highest among the CUD-documented group, followed by the CU-documented group and matched controls. The largest differences between the groups were in the prevalence of anxiety (60.1% CUD-documented, 36.3% CU-documented, 16.8% matched controls), depression (50.3% CUD-documented, 26.3% CU-documented, 12.4% matched controls) and other substance use disorders.

### Adjusted odds of diagnoses

Table 3 shows the adjusted odds that patients with CUD documentation and CU documentation would have diagnoses compared with matched controls and with each other (CUD-documented vs. CU-documented), controlling for alcohol and tobacco diagnoses. For general medical conditions, patients in the CUD-documented group had significantly higher odds of 12 of 13 diagnoses examined when compared to matched controls, with the highest ORs for other substance use disorders (OR = 21.44; 95% CI 9.43–48.73), HIV/AIDS (OR = 7.88; 95% CI 2.58–24.08), and any mental health disorder (OR = 6.99; 95% CI 5.03–9.70). CU-documented patients had higher odds than matched controls of diagnoses for 11 out of 13 diagnoses examined, with the highest odds for other substance use disorders (OR = 3.64; 95% CI 2.53–5.25). Compared to CU-documented patients, CUD-documented patients had significantly higher odds of HIV/AIDS (OR = 6.70; 95% CI 2.10–21.39), other substance use disorders (OR = 5.88; 95% CI 2.43–14.22), and any mental health disorder (OR = 2.48; 95% CI 1.76–3.51).

**Table 3:** Multivariate conditional logistic models on prevalence of health conditions (controlling for alcohol use disorders and tobacco use) odds ratios (95% confidence interval)

	**Cannabis Use Disorder (CUD)-Documented vs Matched Controls**	**Cannabis Use (no disorder) (CU)-Documented vs Matched Controls**	**Cannabis Use Disorder (CUD)-Documented vs Cannabis Use (no disorder) (CU) -Documented**
**General Medical Conditions**			
Cancer	2.00 (1.43–2.80)^**^	1.65 (1.44–1.88)^**^	1.21 (0.85–1.73)
Diabetes Mellitus	1.95 (1.26–3.02)	1.23 (1.01–1.50)	1.58 (0.99–2.53)
Nervous System Disease	2.67 (2.06–3.47)^**^	2.01 (1.81–2.24)^**^	1.33 (1.01–1.75)
Sleep Disorders	3.70 (2.69–5.08)^**^	2.38 (2.07–2.74)^**^	1.55 (1.11–2.17)
Circulatory System Disease	2.04 (1.54–2.69)^**^	1.69 (1.50–1.90)^**^	1.20 (0.90–1.62)
Respiratory Disease	1.58 (1.24–2.00)^*^	1.65 (1.49–1.83)^**^	0.95 (0.74–1.23)
Digestive System Disease	2.08 (1.62–2.67)^**^	2.03 (1.82–2.25)^**^	1.03 (0.79–1.34)
Liver Disease	1.86 (1.20–2.90)^*^	1.68 (1.37–2.06)^**^	1.11 (0.69–1.78)
Musculoskeletal Disease	1.76 (1.36–2.26)^**^	1.67 (1.51–1.85)^**^	1.05 (0.80–1.37)
HIV/AIDS	7.88 (2.58–24.08)^*^	1.18 (0.69–2.00)	6.70 (2.10–21.39)^**^
Sexually Transmitted Disease Other Than HIV/AIDS	3.39 (1.81–6.36)^**^	2.28 (1.68–3.08)^**^	1.49 (0.75–2.94)
Mental Health Disorders	6.99 (5.03–9.70)^**^	2.81 (2.51–3.15)^**^	2.48 (1.76–3.51)^**^
Substance Use Disorder (other than alcohol, cannabis, tobacco)	21.44 (9.43–48.73)^**^	3.64 (2.53–5.25)^**^	5.88 (2.43–14.22)^**^
**Conditions Known To Be Associated With Cannabis**			
Respiratory Disease	1.58 (1.24–2.00)^*^	1.69 (1.05–2.73)	0.95 (0.74–1.23)
COPD	1.67 (1.23–2.27)	1.49 (1.30–1.71)^*^	1.12 (0.81–1.55)
Chronic Obstructive Pulmonary Disease	2.51 (1.47–4.30)^*^	1.57 (1.26–1.95)^**^	1.61 (0.90–2.85)
Cancer	2.00 (1.43–2.80)^**^	1.65 (1.44–1.88)^*^	1.21 (0.85–1.73)
Ischemic Heart Disease	2.51 (1.47–4.30)^*^	1.57 (1.26–1.95)^**^	1.61 (0.90–2.85)
Obstructive Sleep Apnea	2.11 (1.27–3.50)^*^	2.07 (1.64–2.62)^**^	1.02 (0.59–1.75)
Multiple Sclerosis	6.09 (0.64–57.44)	3.14 (1.38–7.12)^*^	1.94 (0.20–18.81)
HIV-AIDS	7.88 (2.58–24.08)^*^	2.28 (1.68–3.08)^**^	6.70 (2.10–21.39)^*^
Tourette Syndrome	0 (0.00–1.00)	2.00 (0.18–22.06)	0 (0.00–1.00)
Testicular Cancer	2.11 (0.25–18.05)	0.70 (0.27–1.86)	3.00 (0.30–29.75)
Chronic Pain	1.85 (1.44–2.38)^**^	1.74 (1.57–1.93)^**^	1.06 (0.81–1.38)
Schizophrenia/Psychotic Disorder	5.98 (2.23–16.03)^*^	1.69 (1.05–2.73)	3.53 (1.18–10.53)
Depression	7.01 (4.79–10.27)^**^	2.46 (2.15–2.81)^**^	2.85 (1.90–4.26)^**^
Anxiety	5.99 (4.37–8.21)^**^	2.74 (2.43–3.08)^**^	2.19 (1.57–3.05)^**^
Bipolar Disorder	6.49 (2.90–14.52)^**^	2.54 (1.84–3.51)**	2.56 (1.07–6.09)
Social Anxiety Disorder	13.03 (2.18–77.94)^*^	1.64 (0.85–3.17)	7.93 (1.24–50.59)
Post-Traumatic Stress Disorder	7.74 (2.66–22.51)^*^	3.41 (1.99–5.83)^**^	2.27 (0.73–7.13)
Substance Use Disorder (other than alcohol, cannabis, tobacco)	21.44 (9.43–48.73)^**^	3.64 (2.53–5.25)^**^	5.88 (2.43–14.22)^*^

^*^P =  < .01 ^**^P =  < .001

Among conditions known to be associated with cannabis, CUD-documented patients had higher odds than matched controls for 14 out of 18 conditions, with the highest ORs for other substance use disorders (OR = 21.44; 95% CI 9.43–48.73), social anxiety disorder (OR = 13.03; 95% CI 2.18–77.94), HIV/AIDS (OR = 7.88; 95% CI 2.58–24.08), post-traumatic stress disorder (OR = 7.44; 95% CI 2.66–22.51), depression (OR = 7.01: 95% CI 4,79–10.27), any mental health disorder (OR = 6.99; 95% CI 5.03–9.70), and bipolar disorder (OR = 6.49: 95% CI 2.90–14.52). CU-documented patients had higher odds of 13 out of 18 conditions compared to matched controls, with the highest ORs for other substance use disorders (OR = 3.64; 95% CI 2.53–5.25) and post-traumatic stress disorder (OR = 3.41; 95% CI 2.53–5.25). CUD-documented patients were significantly more likely than CU-documented patients to have HIV/AIDS (OR = 6.70; 95% CI 2.10–21.39), other substance use disorder (OR = 5.88; 95% CI 2.42–14.22), depression (OR = 2.85; 95% CI 1.90–4.26), and anxiety (OR = 2.19: 95% CI 1.57–3.05).

## Discussion

### Key results

Under one percent of the sample in this study had documentation of CU or CUD in their EHR, compared to studies of EHR data from Washington State, which found EHR-documented cannabis use rates between 15 and 22% [16, 17]. Some of this difference may be due to higher levels of adult CU, frequent cannabis use, and CUD in states like Washington that allow non-medical marijuana use [23]. Also, the sample in this study was mostly commercially insured and of relatively high income, so these factors may account for the differences from the Washington State samples. However, the rate of CU and CUD documentation in EHRs in this study was still surprisingly low. According to the U.S. National Survey on Drug Use and Health, 16.4% of Californians over age 12 reported past-year CU between 2014 and 2017, and 2.0% of this population had a CUD [31]. Part of the reason for these discrepancies could be in the methods used to identify CU and CUD among the patient populations. In the Washington State studies, all patients completed a cannabis screening at a primary care visit, whereas in this study, patients were not routinely screened. The large gap between rates of CU and CUD in California population surveys and the frequency of CU and CUD documentation in EHRs in this study could be indicators of how cannabis use can go undetected during primary care visits in the absence of systematic screening [32]. California healthcare providers may now begin screening for CU and CUD more since the U.S. Preventive Services Task Force has recommended drug use screening for adults in primary care [33], and this may lead to better identification of cannabis use in medical settings. This finding also underscores the importance of having healthcare providers in other states and countries—both those with and without legalized marijuana—systematically screen patients for CUD and cannabis use.

Study findings also shed light on the association between CUD documentation, CU documentation, and health among primary care patients in medical marijuana jurisdictions, showing that cannabis use is associated with many physical health conditions. CUD-documented patients in this study were over seven times as likely as matched controls to have HIV/AIDS diagnoses, three times as likely to have sleep disorder diagnoses, and twice as likely to have nervous system disease, digestive system disease, circulatory system disease, ischemic heart disease, fibromyalgia, and sleep apnea diagnoses. CU-documented patients also had increased odds for most of these conditions, though not as much as CUD-documented patients. Elevated rates of medical problems could result from direct physical effects of regular cannabis use and associated behaviors, and the fact that individuals with substance use disorders (including cannabis use disorder) are less likely than others to access and receive quality health care [10, 34–36]. Conversely, it is possible that some of this association is due to people with medical conditions using cannabis to manage or alleviate their symptoms [13, 37–39]. These findings indicate a stronger association between cannabis use and medical diagnoses than that found by Matson et al. [17] in their study of EHR data from Washington State. These differences may be attributable to the fact that unlike Washington State, California was a medical marijuana state at the time of this study, but had not yet legalized cannabis use generally. California patients may have been less likely to report their cannabis use due to fear of legal or social consequences for disclosing their substance use, leading providers to only identify CU or CUD in cases where it was discernible from patient presentation. Furthermore, unlike in the Matson et al. study, patients in this sample were not identified by universal screening. Consequently, it is possible that patients in this sample only had their cannabis use noted in their EHR if it emerged as a topic in the course of their primary care encounter, meaning that their cannabis use and its consequences may have been particularly severe or salient. By having respectful, nonjudgmental, and balanced discussions about the pros and cons of cannabis use with patients, medical providers may be able to decrease patient reluctance to disclose and discuss their cannabis use [5, 40]. Further research can help determine the degree to which the policy context and/or different methods for identifying patients who used cannabis may have accounted for the different findings reported here and those reported in other EHR studies.

When compared to physical health diagnoses, the odds of CU-documented and CUD-documented patients having behavioral health diagnoses relative to matched controls were particularly high. CUD-documented patients were nearly six times as likely as matched controls to have diagnoses of schizophrenia/psychotic disorders, over seven times as likely to have a depression diagnoses, over six times has likely to have a bipolar disorder diagnosis, and six times as likely to have anxiety diagnoses. For CU-documented patients, odds of these conditions were also elevated, but not nearly as much as for CUD-documented patients. Moreover, CUD-documented patients were over three times as likely as CU patients to have schizophrenia/psychotic disorders, and over twice as likely to have depression or anxiety diagnoses. These findings support the extensive body of research demonstrating a correlation between cannabis use and mental health problems [8, 11, 16, 41–44] and the association between cannabis use documentation and the presence of psychiatric diagnoses in EHRs [15, 17]. They also underscore the importance of screening and assessment for co-occurring mental health disorders among people who use cannabis or have cannabis use disorders, and ensuring that they receive evidence-based psychosocial and pharmacological interventions as needed [45–48]. Many of the behavioral interventions that have shown efficacy in addressing problematic cannabis use—such as motivational enhancement therapy, cognitive behavioral therapy, and contingency management—are also effective for treating other behavioral disorders, and could help improve the overall behavioral health of primary care patients with CUD [46, 47, 49].

Odds of other substance use disorders were also higher among the CUD-documented group when compared to matched controls, as they had over 21 times the risk of having other substance use disorder diagnoses. As with mental health diagnoses, the CU-documented group was also at elevated risk for substance use disorder diagnoses, but not nearly as much as the CUD-documented group. Compared to CU-documented patients, CUD-documented patients were over five times as likely to have another substance use disorder diagnosis. These findings align with other research demonstrating associations between cannabis use, increased use of other substances, and increased risk for other substance use disorders [8, 16, 17, 38, 50, 51].

The significantly increased odds of HIV/AIDS diagnoses among CUD-documented patients—but not among CU-documented patients—is also notable. There is limited evidence showing cannabis can be effective for increasing appetite and decreasing weight loss associated with HIV/AIDS [13], though evidence on its long-term safety and impact on long-term AIDS-related morbidity and mortality is limited [52]. However, some research has found cannabis dependence is associated with lower adherence to antiretroviral therapy and increased HIV symptoms and medication side effects [53], so the high prevalence of HIV/AIDS among the CUD-documented group in this study is concerning. This finding aligns with previous research showing relatively high levels of frequent cannabis use among people living with HIV [54], and highlights the need to detect CUD among this population and provide them with effective counseling and support to help them manage their cannabis use [55].

### Limitations

Several key limitations should be noted. First, the study drew data from one university health system, and may not be generalizable to other primary care populations in other regions or countries. Second, measures of CUD and CU documentation were extracted from EHRs that did not have specific questions prompting provider to elicit data concerning cannabis use. This could account for the low rates of CUD and CU documentation in the sample, and it is possible that CUD and CU were only noted either when patients mentioned cannabis, or when providers detected issues that prompted them to ask about substance use. Consequently, there is a good possibility that only patients with outward signs of cannabis use or who self-disclosed cannabis use were detected, and these patients may use cannabis more frequently or heavily than most patients who use the drug. Other studies have documented under-diagnosis of CUD in medical records in the absence of routine screening and assessments [32]. Study findings can be interpreted as supporting the associations between CUD and CU at a threshold level that merits documentation in medical records, and should be interpreted within this context.

Third, since the dataset only allowed for identification of CUD and CU documentation, the study does not include information concerning frequency of use, duration of use, quantities used, types of products used, or potencies of cannabis products consumed. This information would be needed in order to come to more precise conclusions concerning the relationship between cannabis and medical conditions. Despite these limitations, the medical records data used have several advantages, including the large and diverse population, standardized medical codes and detailed case notes, and accumulation of relevant diagnostic data over multiple visits.

## Conclusions

This study highlights the relationship between cannabis use and cannabis use disorder documentation with other medical, mental health, and other substance use disorders in states where medical marijuana is legal. Given the strong associations of cannabis use and cannabis use disorder documentation with health problems—particularly those related to mental health, substance use, and HIV—it is important for healthcare providers in such jurisdictions to be prepared to identify cannabis use and cannabis use disorders, and address these comorbidities among these patients. Study findings also suggest the need for future research on optimal strategies for initiating discussion of cannabis use and its potential benefits and adverse effects with primary care patients, particularly those already known to have medical and behavioral health conditions.

## Data Availability

Data for this study came from medical system EHRs and is not publicly available. These data are only available to clinicians and researchers from the University of California, Los Angeles.

## Appendix

### ICD-10 codes used to identify medical and behavioral health conditions

Cancer: C00-26, C30-41, C43-58, C60-76, D00-09, D37-49

Diabetes Mellitus: E08-E13

Nervous System Disease: G00-G99

Sleep Disorders: F10.182, F10.282, F11.182, F11.282, F11.982, F13.182, F13.282, F13.982, F14.182, F14.282, F14.982, F15.182, F15.282, F15.982, F19.182, F19.282, F19.982, F51, G47

Circulatory System Disease: I00-I99

Respiratory Disease: J00-J99

Digestive System Disease: K00-K98

Liver Disease: K70-77

Musculoskeletal Disease: M00-M99

HIV/AIDS B20, Z21

Sexually Transmitted Diseases other than HIV/AIDS: A50-64

Mental Health Disorders: F06, F20-48

Alcohol Use Disorder: F10

Tobacco Use Disorder: F17

Substance Use Disorder other than alcohol, cannabis, tobacco: F11, F13-16, F18-19

Chronic Obstructive Pulmonary Disease: J40-47

Ischemic Heart Disease: I20-25

Obstructive Sleep Apnea: G47.33

Multiple Sclerosis: G35

Tourette Syndrome: F95.2

Testicular Cancer: C62

Chronic Pain: F45.4, G43, G44.2, G89, M00-99

Schizophrenia/Psychotic Disorder: F06.0, F06.2, F20-29

Depression: F06.31-32, F32, F33, F34.1

Anxiety: F06.4, F40-48

Bipolar Disorder: F31

Social Anxiety Disorder: F40.10

Post-Traumatic Stress Disorder: F43.10, F43.12

## Abbreviations

EHR: Electronic Health Record
CUD: Cannabis Use Disorder
CU: Cannabis Use
ICD-10: International Classification of Diseases, Tenth Revision
HIV/AIDS: Human Immunodeficiency Virus/Acquired Immunodeficiency Syndrome
COPD: Chronic Obstructive Pulmonary Disease
OR: Odds Ratio
US: United States
CI: Confidence Interval
